# Diverse Small Molecule Inhibitors of Human Apurinic/Apyrimidinic Endonuclease APE1 Identified from a Screen of a Large Public Collection

**DOI:** 10.1371/journal.pone.0047974

**Published:** 2012-10-23

**Authors:** Dorjbal Dorjsuren, Daemyung Kim, Vaddadi N. Vyjayanti, David J. Maloney, Ajit Jadhav, David M. Wilson, Anton Simeonov

**Affiliations:** 1 NIH Chemical Genomics Center, National Center for Advancing Translational Sciences, National Institutes of Health, Bethesda, Maryland, United States of America; 2 Laboratory of Molecular Gerontology, National Institute on Aging, National Institutes of Health, Baltimore, Maryland, United States of America; 3 Department of Genetic Engineering, Cheongju University, Cheongju, Republic of Korea; Northwestern University Feinberg School of Medicine, United States of America

## Abstract

The major human apurinic/apyrimidinic endonuclease APE1 plays a pivotal role in the repair of base damage via participation in the DNA base excision repair (BER) pathway. Increased activity of APE1, often observed in tumor cells, is thought to contribute to resistance to various anticancer drugs, whereas down-regulation of APE1 sensitizes cells to DNA damaging agents. Thus, inhibiting APE1 repair endonuclease function in cancer cells is considered a promising strategy to overcome therapeutic agent resistance. Despite ongoing efforts, inhibitors of APE1 with adequate drug-like properties have yet to be discovered. Using a kinetic fluorescence assay, we conducted a fully-automated high-throughput screen (HTS) of the NIH Molecular Libraries Small Molecule Repository (MLSMR), as well as additional public collections, with each compound tested as a 7-concentration series in a 4 µL reaction volume. Actives identified from the screen were subjected to a panel of confirmatory and counterscreen tests. Several active molecules were identified that inhibited APE1 in two independent assay formats and exhibited potentiation of the genotoxic effect of methyl methanesulfonate with a concomitant increase in AP sites, a hallmark of intracellular APE1 inhibition; a number of these chemotypes could be good starting points for further medicinal chemistry optimization. To our knowledge, this represents the largest-scale HTS to identify inhibitors of APE1, and provides a key first step in the development of novel agents targeting BER for cancer treatment.

## Introduction

The genome of mammalian cells is under constant threat from both endogenous (namely reactive oxygen species, such as the superoxide anion, hydroxyl radical, hydrogen peroxide, and nitrogen-reactive species) and exogenous (*e.g.,* sunlight, ionizing radiation, chemical compounds and genotoxic drugs) DNA damaging agents that can introduce mutagenic and cytotoxic DNA lesions [1], [2]. For example, it has been estimated that spontaneous depurination events result in more than 10,000 abasic lesions per mammalian cell per day [3], [4]. Left unrepaired, DNA damage can result in detrimental biological consequences to the organism, including cell death and mutations that drive transformation to malignancy. Cells use various DNA repair systems as defenses to protect their genomes from DNA damaging agents and to maintain genome stability [5], [6], [7]. Not surprisingly, cells with a defect in one of their DNA repair mechanisms are typically more sensitive to certain genotoxic agents and suffer increased mutagenesis.

Most antitumor drugs (*e.g.,* alkylating, cross-linking and intercalating agents, topoisomerase inhibitors, and certain anti-metabolites) induce DNA lesions that ultimately block or interfere with DNA replication in rapidly dividing cancer cells, resulting in increased susceptibility to activation of various programmed cell death responses [8]. An elevated DNA repair capacity in tumor cells results in anticancer drug and radiation resistance, severely limiting the efficacy of these agents. Recent basic and clinical studies have demonstrated emerging concept designs to block the functions of various proteins in specific DNA repair pathways, which would sensitize cancer cells to DNA damaging agents and potentially lead to an improved therapeutic outcome [9], [10].

The base excision repair (BER) pathway is responsible for correcting damage to single DNA bases or to the sugar moiety of the phosphodiester backbone. Typically, the BER process starts with the enzymatic removal of a damaged base by either a mono- or a bi-functional DNA glycosylase, which creates an abasic (AP) site or in some instances a DNA strand break. The AP site is incised by an essential enzyme known as apurinic/apyrimidinic endonuclease-1 (APE1) [11], which generates a single-stranded gap in DNA with 3′-hydroxyl and 5′-deoxyribosephosphate termini. This gap is filled in and ultimately sealed by the concerted action of DNA polymerases and ligases [4]. In mammalian cells, APE1 is responsible for at least 95% of the endonuclease activity that incises at abasic sites as part of the short-patch and long-patch BER subpathways. APE1 has been found not only to be required for animal viability, as deletion of both alleles of the *APE1* gene in mice leads to embryonic lethality, but also for cell viability in culture [12], [13].

Elevated levels of APE1 have been found in medulloblastoma and primitive neuroectodermal tumors, prostate cancers, head-and-neck cancers, non-small cell lung carcinomas, gliomas, and osteosarcomas [4]. Over-expression of APE1 has been correlated with increased cellular resistance to chemotherapeutic agents. Moreover, APE1-deficient cells exhibit hypersensitivity to methyl methanesulfonate (MMS), hydrogen peroxide, bleomycin, temozolomide, gemcitabine, 1,3-bis (2-chloroethyl)-1-nitrosourea (a.k.a. *Carmustine*), and the nucleoside analogue ß-L-dioxolane-cytidine (a.k.a. *Troxacitabine*) [4], [14]. Furthermore, the expression of a dominant-negative APE1 protein (termed ED), which binds with high affinity to substrate DNA and blocks subsequent repair steps [15], augments the cell killing effect of 5-fluorouracil and 5-fluorodeoxyuridine, implicating BER in the cellular response to such anti-metabolites [16]. These data indicate that APE1 is an attractive and rational target in the effort to improve therapeutic efficacy of clinical DNA-interactive drugs through the inactivation of the critical BER pathway.

A significant limitation of anti-cancer cytotoxins is their harmful side-effects on normal tissue. While combinatorial treatment strategies are still of interest, researchers and clinicians have been pursuing the idea of synthetic lethality to reduce potential off-target toxicities. In this scenario, inhibition of two independent processes separately has little cellular consequence, whereas inactivation of both pathways simultaneously leads to cell death. This model has been exploited in the case of cancers deficient in the breast cancer-related homologous repair proteins, BRCA1 and BRCA2. Here, inhibitors against the poly(ADP-ribose) polymerase protein, PARP-1, which operates in strand break responses, including the single-strand break repair sub-pathway of BER, have been shown to induce selective cell killing of BRCA-deficient cells [8], [17], presumably due to replication fork collapse and increased genetic instability. Relevant to the effort within, inhibitors against APE1 have been found to be synthetically lethal to cells deficient in BRCA1 or BRCA2, or the checkpoint signaling protein ATM, inducing accumulation of DNA double-strand breaks as well as G2/M cell cycle arrest [18].

A number of chemical libraries have been screened to identify small molecule inhibitors of APE1 endonuclease activity [19], [20], [21], [22], [23]. Several molecules were identified from these efforts, including 7-nitro-indole-2-carboxylic acid identified from a screen of a 5000-compound collection [19]; several arylstibonic acid derivatives identified from a screen of the National Cancer Institute Diversity Set [20]; Reactive Blue 2, 6-hydroxy-DL-DOPA, and myricetin, reported as prioritized hits from a screen of the LOPAC^1280^ collection of bioactive compounds [21]; and 2,4,9-trimethylbenzo [b] [1], [8] naphthyridin-5-amine (dubbed AR03), selected as the top hit from a 60,000-member library screen [23]. Additionally, an *in-silico* screen based on a pharmacophore approach has led to the identification of several APE1 inhibitors sharing a hydrophobic middle segment to which at least two carboxyl substituents (or other negatively charged groups) are attached via a range of linkers [22]; however, APE1 inhibition has not been demonstrated for these compounds in cell-based models. At present, none of the above compounds has been shown to have clinical utility and, with very few exceptions, the inhibitors reported to date are not readily amenable to further optimization by medicinal chemistry due to multiple liabilities stemming from their chemical structure [24].

We describe herein the first small molecule inhibitors of human APE1 identified by quantitative high-throughput screening (qHTS) [25] of a large public compound collection, the Molecular Libraries Small Molecule Repository (MLSMR) of >300,000 compounds, as well as additional public libraries of the NIH Chemical Genomics Center. Prioritized hits were further characterized by a panel of biochemical assays and in MMS cell toxicity potentiation models. Select compounds were also tested in an AP site measurement assay designed to ascertain the inhibitors’ effect on APE1 within a cellular context.

**Figure 1:**
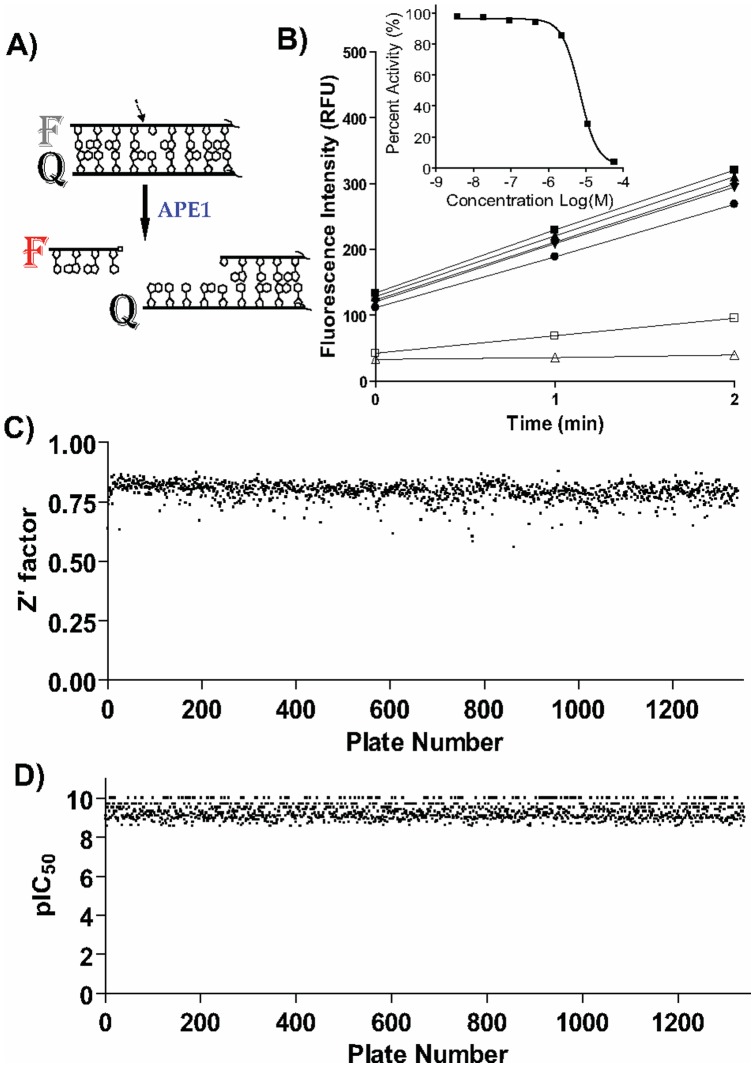
High-throughput screen. A) Assay principle. APE1 catalyzes and incision 5′ relative to the abasic site analog (THF) to liberate a short 5′-fluorophore donor F-labeled deoxyoligonucleotide, causing increased fluorescence signal. F represents TAMRA fluorophore and Q represents Black Hole Quencher 2. The APE1 incision site is indicated by the arrow. B) High-speed data collection allows monitoring of the reaction progress in kinetic mode as shown in the main panel (3 data points collected over the course of 2 min, shown for 7 wells representing the serial dilution of library compound MLS000090966); the changes in fluorescence signal for each well over the two-minute period are normalized against no-enzyme and no-inhibitor controls to produce the concentration response curve for the sample as shown in the inset. C) A stable Z’ screening factor was maintained throughout the screen. D) A dilution series of the previously reported arylstibonic acid inhibitor NSC-13755 applied to every assay plate yielded a near-constant IC_50_ of 35 nM.

## Materials and Methods

### Reagents

Dimethyl sulfoxide (DMSO, certified ACS grade) was purchased from Thermo Fisher Scientific. Tris-HCl, Thiazole Orange (ThO), Tween-20, NaCl, and MgCl_2_ were obtained from Sigma, while the arylstibonic control inhibitor NSC-13755 was supplied by the National Cancer Institute Developmental Therapeutics Program Repository.

**Figure 2:**
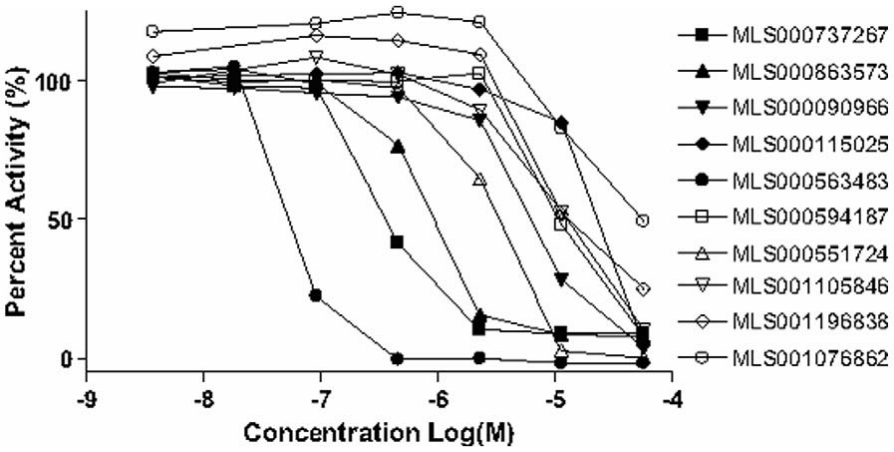
Representative curves observed from 10 screening hits chosen to demonstrate the range of potencies observed in the concentration-response-based screen. Structures and additional data associated with these hits are presented within Figure 7.

The enzymes and substrates employed in this study (human APE1 and *E. coli* Endo IV enzymes, fluorogenic and radioassay substrates) were prepared and characterized as described previously [21]. The fluorescent probe employed in the fluorescence polarization (FP) displacement assay was of the same composition as the fluorogenic HTS substrate with the exception of the Black Hole Quencher-2 moiety. The double-stranded 17-mer DNA fragment used in the ThO DNA binding assay was of the same sequence as the fluorogenic HTS substrate but carried no dye labels.

**Figure 3:**
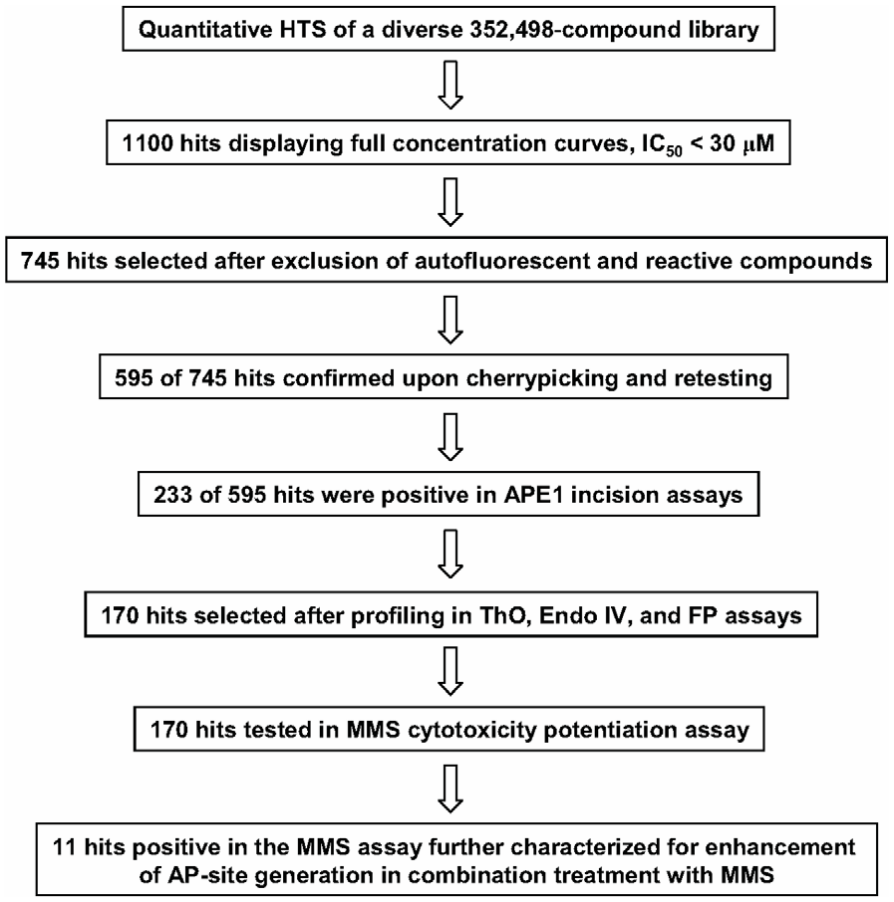
Hit progression flow chart.

**Figure 4:**
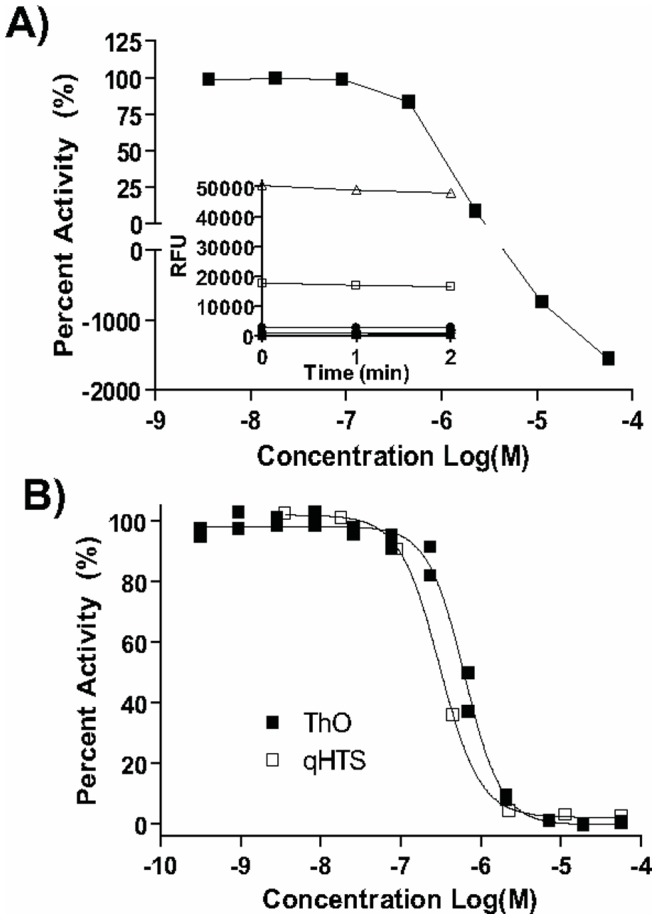
Elimination of assay artifacts and promiscuous hits. A) An autofluorescent compound contributes fluorescence intensity well in excess of the assay reaction’s average leading to the computation of aberrant concentration response curve. B) An example of a promiscuous hit acting by strong DNA binding as evidenced by the highly similar concentration responses observed in the primary screen (empty squares) and the ThO counterscreen (filled squares).

### Compound Library

The 352,498-member library comprised two main subsets: NIH Molecular Libraries Small Molecule Repository (MLSMR), prepared as 10 mM stock solutions in 384-well plates and delivered by Biofocus DPI (South San Francisco, CA, http://mli.nih.gov/mli/compound-repository/mlsmrcompounds/), and NCGC internal exploratory collection, which consisted of several commercially available libraries, as well as collections from academic compound libraries. The compound library was sourced as DMSO solutions at initial concentrations ranging between 2 and 10 mM and was further serially diluted for qHTS in 1536-well format as described in detail elsewhere [26], [27]. For follow-up testing of primary screen actives, screening hits and their analogues were sourced as powders from the respective original suppliers (Sigma-Aldrich, NCI, Asinex, ChemBridge, Tocris, Ambinter, and ChemDiv), dissolved in DMSO to produce 10 mM initial stock solutions, and serially diluted in twofold steps for a total of 12 concentrations in duplicate. The identities of all compounds screened are available in PubChem under Assay Identifier 2517, http://pubchem.ncbi.nlm.nih.gov/.

**Figure 5:**
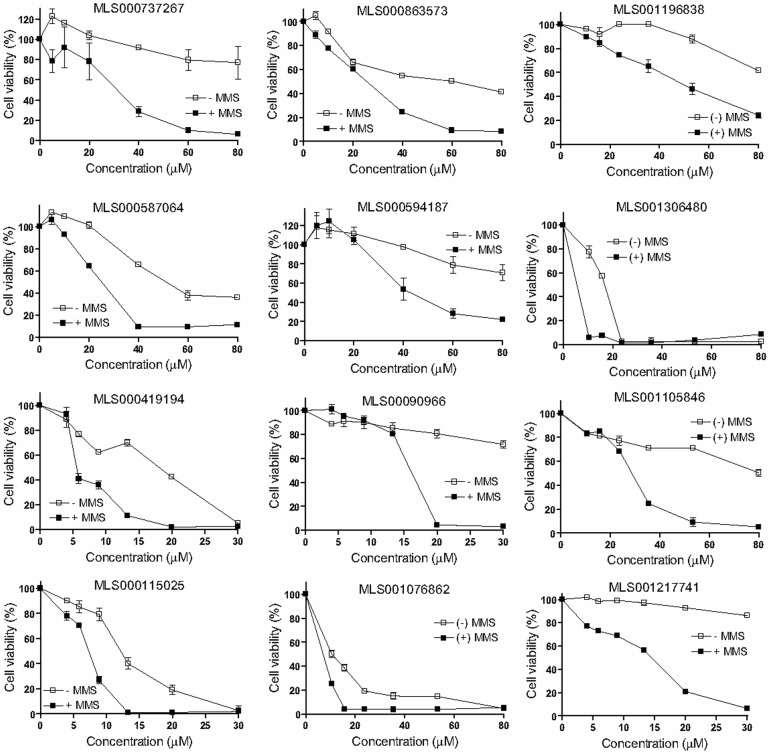
Significant potentiation of the genotoxic effect of MMS by 12 prioritized hits (designated P in Table S1). HeLa cells were exposed to a dilution series of each compound shown in the absence (empty squares) and presence of 400 µM MMS (filled squares), and after a 24-hour incubation the cell viability was measured by ATP-content detection using CellTiter Glo. Results are presented as averages and standard deviations from duplicate samples, normalized against control.

**Figure 6:**
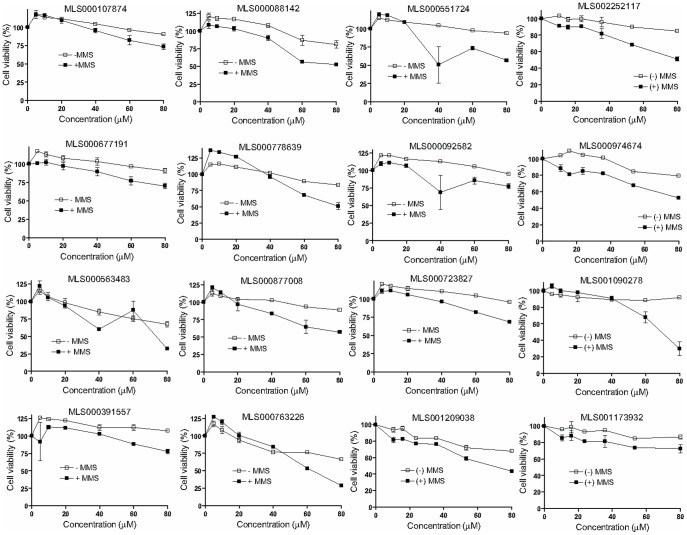
Modest potentiation of the genotoxic effect of MMS exhibited by 16 prioritized hits (designated I in Table S1). HeLa cells were exposed to a dilution series of each compound shown in the absence (empty squares) and presence of 400 µM MMS (filled squares), and after a 24-hour incubation the cell viability was measured by ATP-content detection using CellTiter Glo. Results are presented as averages and standard deviations from duplicate samples, normalized against vehicle control.

**Figure 7:**
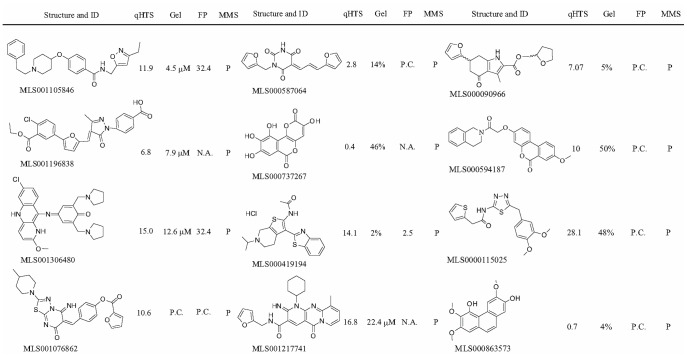
Screening hits showing significant activity in the MMS cytotoxicity enhancement experiments. qHTS, IC_50_ (µM) obtained in the initial quantitative high-throughput screen; Gel, percent incision observed in the presence of 100 µM compound using the radiotracer detection or estimated IC_50_ value (µM) using the fluorescence detection; FP, IC_50_ (µM) or annotation of response (N.A., no activity observed; P.C., partial concentration response curve) obtained in the fluorescence polarization displacement assay; MMS, potentiation of the genotoxic effect of methylmethane sulfonate (P, positive).

### High-throughput Screen

The screen was performed following the previously published protocol [21]. All screening operations were performed on a fully integrated robotic system (Kalypsys, San Diego, CA) [28] with library plates screened proceeding from the lowest to the highest concentration to minimize compound carryover [26]. Vehicle-only plates, with DMSO being pin-transferred to the entire column 5–48 compound area, were included regularly throughout the screen in order to record any systematic shifts in assay signal. During the screen, reagent bottles were kept at 4°C and all liquid lines were covered with aluminum foil to minimize degradation.

**Figure 8:**
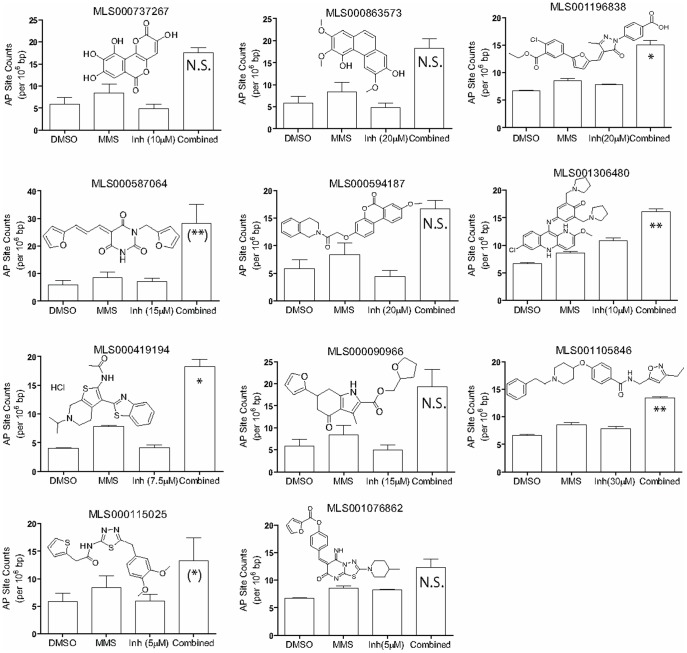
AP-site accumulation. HeLa cells were exposed to each compound shown in the absence and presence of 275 µM MMS, and total genomic AP sites were measured as described in Methods. The number of AP sites per 10^6^ base pairs of genomic DNA are presented as the average and standard deviation of two independent measurements. P-values (*P<0.05, **P<0.01) were calculated to evaluate the significance of the enhancement of AP site levels produced by the combined treatment versus MMS alone. N.S., not significant (P≥0.05); P-value designations for MLS000587064 and MLS000115025 shown in parentheses are derived from the repeat testing reported in Figure S1.

Screening data were corrected and normalized, and concentration–effect relationships was derived using in-house developed algorithms [25]. Percent activity was computed after normalization using the median values of the uninhibited enzyme control (32 wells located in column 1) and the no-enzyme, or 100% inhibited, control (64 wells, entire columns 3 and 4), respectively, and concentration-response data were fitted using a four parameter Hill equation [29] by minimizing the residual error between the modeled and observed responses. After curve fitting, active compounds were analyzed based on their potency and concentration-response curve characteristics, taking into consideration the presence of asymptotes, efficacy of response, and confidence of curve fit [25]. After preliminary clustering of actives based on structural similarity analysis using Leadscope software (Leadscope Inc., Columbus, OH) [30] selected hits were procured for re-testing in the primary screening assay and potential follow-up studies.

### ThO DNA Displacement and *E. coli* Endo IV Profiling Assays

In order to screen out nonselective compounds, such as non-specific DNA binders and inhibitors of related enzymes, we used a miniaturized ThO DNA displacement assay and a counterscreen against the bacterial endonuclease *E. coli* Endo IV, respectively, following the previously published protocols [21].

### Fluorescence Polarization (FP) Displacement Assay

Double-stranded oligodeoxynucleotide containing tetrahydrofuran (THF) abasic site labeled with TAMRA at the 5′-end was used as the labeled binding probe. Inhibition of APE1 binding to labeled probe was detected by a decrease in the fluorescence polarization (FP) of the fluorophore. Briefly, 4 µL mixture of 20 nM APE1 and 5 nM probe contained in the enzymatic assay buffer from which Mg^2+^ was omitted, was dispensed into 1536-well Greiner black solid bottom plates. Compounds (23 nL) were transferred via pintool, and after a 15-minute incubation at room temperature, fluorescence polarization was measured in a ViewLux High-throughput CCD imager (480 nm excitation and 540 nm emission) to determine the degree of probe displacement caused by the test compounds.

### Gel-based APE1 Assays

To test the compounds’ inhibitory activity after the primary screen, two assays designed to monitor the cleavage of substrate to product through electrophoretic separation were utilized. The radiotracer incision assay, utilizing a ^32^P-labeled substrate, was performed essentially as described [21], [31], [32]. Incision reactions utilizing ^32^P-labeled substrate were carried out for 5 min at 37°C. After the addition of an equal volume of stop buffer (0.05% bromophenol blue and xylene cyanol, 20 mM EDTA, 95% formamide), the substrate and product were separated on a standard polyacrylamide denaturing gel and quantified by PhosphorImager analysis [32]. The fluorescence-based assay utilized a 5′-TAMRA labeled 32-mer oligonucleotide containing a THF moiety at position 18 (5′-TAMRA-TTTTTTTTTTTTTCACCFTCGTACGACTCCGT-3′) annealed with a complementary 20-mer oligonucleotide (5′-ACGGAGTCGTACGAGGGTGA-3′) was used in this assay. APE1 cleavage of the substrate results in a 18-mer TAMRA-labeled fragment which was separated by a non-denaturing polyacrylamide gel electrophoresis and quantitated in a Gel Doc XR imaging system, BioRad (Hercules, CA). The advantage of this assay is that it can be used in a relatively high throughput mode with the inhibitors tested at a range of concentrations (0.17 µM to 125 µM was used here). APE1 (0.15 nM) was added to a reaction mixture of 20 µL containing assay buffer (40 mM HEPES pH 7.5, 50 mM NaCl, 1 mM MgCl_2_ and 2 mM DTT), and labeled ds DNA substrate (final 75 nM). The mixture was incubated at RT for 15 min, and the reaction was stopped by the addition 20 µL of 2X dye stop solution (10% glycerol, 20 mM EDTA and bromophenol blue). Then 10 µL of the mixture was resolved on 20% polyacrylamide gel in 1X Tris-borate EDTA running buffer at 250 V for 40 min.

### MMS Potentiation Assay

HeLa cells were plated at 6000 cells/well in DMEM culture medium containing 10% FBS into white solid bottom 384-well cell culture plates. The plates were incubated at 37°C overnight for cell attachment, followed by media replacement. The fresh medium contained serial dilutions of compounds of interest in the presence (0.4 mM final) or absence of MMS. The plates were incubated for 24 h at 37°C. Cell viability was determined by luminescence detection after an addition of 15 µL of CellTiter Glo reagent (Promega, Madison, WI). Percent viability was calculated for each concentration of compound tested in duplicate by relating the corresponding luminescence to that of a negative DMSO vehicle control. MMS potentiation trends were defined as follows: negative (-), if cell viability in the compound-plus-MMS treatment was the same as that of the DMSO-plus-MMS control (overlapping dose-response curves); inconclusive (I), if the shift in the cell viability dose-response upon MMS inclusion relative to compound-alone was less than 30%; and positive (P), if the difference between the compound-plus-MMS and compound-alone curves was at least 30%, and maintained within at least a two-fold compound concentration span.

### AP Site Measurement

HeLa cells at 80% confluency (6×10^5^ cells) were subjected to the following treatments in a 6-well plate for 24 hours at 37°C: 1) DMSO alone, 2) 275 µM MMS, 3) APE1 inhibitors dissolved in DMSO (5–20 µM final compound concentration), and 4) the inhibitors as in treatment 3 in combination with 275 µM MMS. Cells were then harvested and processed using a Qiagen Genomic DNA isolation kit (Germantown, MD). After DNA quantitation, AP sites were measured using the DNA Damage Quantification kit from Dojindo Molecular Technologies (Rockville, MD).

## Results and Discussion

### Quantitative High-throughput Screen

To screen for inhibitors of APE1 incision activity, we employed a purified-enzyme biochemical assay with a model substrate featuring a red-shifted fluorescent reporter in combination with a dark non-emitting quencher. These moieties were incorporated as end labels onto a 17-mer double-stranded DNA fragment containing a centrally-located tetrahydrofuran AP site sugar mimetic, which serves as the APE1 cleavage site. Upon APE1-catalyzed strand scission immediately 5′ to the AP site analog, the shortened oligodeoxynucleotide carrying the fluorescent reporter spontaneously dissociates from the rest of the substrate, leading to an increase in fluorescence due to its spatial separation from the dark quencher (Figure 1A). Further details on the generation and validation of this substrate in fully-integrated robotic miniaturized assays have been reported elsewhere [21]. Prior to the full-collection screen, the assay was tested and found to perform reproducibly by screening the LOPAC^1280^ library of pharmacologically active compounds in triplicate using a fully-integrated robotic system (data not shown).

The assay was applied to screen a diverse 352,498-compound library that contained the National Institutes of Health public collection, the MLSMR. The compounds tested were arrayed as seven-point titrations, at final concentrations of 57 µM, 11.4 µM, 2.3 µM, 457 nM, 91 nM, 18 nM, and to 3.7 nM. Using a high-speed whole-plate fluorescence imager, the assay data for the entire screen was conducted in kinetic mode, with the APE1 incision reaction being monitored over the initial linear time frame of 2 min (Figure 1B). Thus, any inhibition associated with each sample was computed from the alteration in fluorescence intensity over the time-course measurement period, after normalization against the appropriate controls (Figure 1B, inset). The assay performed well during the entire course of the screen: the Z’ statistical factor remained consistent without fluctuation, at an average of 0.79 (the maximum Z’ factor possible is 1.0, with values of greater than 0.5 being considered an indication of a highly stable screening assay) [33] (Figure 1C). In addition, the intra-plate control titration of the arylstibonic inhibitor NSC-13755 [20] yielded a near-constant concentration-response curve with an average IC_50_ of 35 nM and a minimum significant ratio of 1.9 (the best possible ratio is 1.0, while ratios of less than 4 are generally indicative of an assay with high test-to-test reproducibility of dose responses) [27], [34] (Figure 1D).

Unlike traditional HTS, qHTS provides a concentration response curve (CRC) for each compound and allows for calculation of an IC_50_ value for each compound in the primary screen. Approximately 1,100 compounds with full concentration-response curves and IC_50_ values of less than 30 µM were identified, and similarity analysis of the hits led to 121 clusters and 154 singletons, representing a wide variety of structural classes (the qHTS results are available in PubChem under Assay Identifier 2517, http://pubchem.ncbi.nlm.nih.gov/). Representative concentration-response curves from 8 hits spanning most of the potency range (double-digit nanomolar to double-digit micromolar IC_50_) are shown in Figure 2. The progression of hits through the respective steps of cheminformatics analysis, confirmatory testing, and additional profiling, is depicted as a flow chart in Figure 3.

After exclusion of heavy metal- and reactive functionality-containing molecules, and after using the real-time kinetic screening data to flag compounds that interfere with the assay signal by contributing excessive amounts of fluorescence [35] (an example of a highly fluorescent hit, the bioactive fluorescent sensor calcein NCGC00094849, is shown in Figure 4A), 745 hits were selected for further characterization based on potencies and concentration-response curve quality. Of the 745 cherry-picked compounds, 595 (80%) exhibited activity upon retesting using the original fluorogenic screening assay.

### Follow-up Testing of Primary Screening Hits

To eliminate false positive hits, all 595 confirmed molecules were tested for their ability to inhibit APE1 incision activity using biochemical assays that involve electrophoretic separation of the substrate and cleavage product. We adopted a two-step approach: (1) hits possessing complete screen-derived concentration response curves were tested at a single concentration in the low-throughput electrophoretic separation assay with radiolabel detection and (2) lower confidence hits possessing either incomplete or noisy concentration response curves were tested as a seven-point dilution series using a higher-throughput electrophoretic separation assay with fluorescence detection. Of the 391 compounds tested in the radioassay, 112 displayed at least 50% inhibition of APE1 activity at 100 µM. Given that the radioassay was specifically conducted at a substrate conversion rate approaching 100%, the fact that a majority of the HTS hits (namely, the weakest and most nonspecific) failed to pass this rigorous APE1 inhibition criterion was not unexpected. Of the 204 compounds tested in the fluorescence-based gel assay, 111 displayed reproducible dose-dependent inhibition. A total of 223 positive compounds showing activity in these electrophoretic separation based assays were then subjected to a panel of assays in order to further assess their engagement with the APE1 target *in vitro*, as well as to evaluate their selectivity. The complete set of results obtained for these 223 compounds in the below tests is provided within Table S1.

To detect screening hits that inhibit APE1 activity through non-specific DNA interactions, we employed a previously established miniaturized ThO dye displacement assay [21]. Forty-three compounds were active in the DNA-binding counter-screen; the majority of these compounds were weak DNA binders (Table S1). Most of the DNA binders possessed the typical chemical features associated with DNA binding: (i) extended conjugated unsaturated ring systems, which would allow them to intercalate between the stacked bases, and/or (ii) accumulation of positively-charged nitrogens, which would permit nonspecific electrostatic interactions with DNA. Of note, classic DNA binders, such as daunorubicin- and tetracycline-like compounds (for example NCGC00093976, NCGC00024246, and NCGC00163605, Table S1), exhibited a strong dose-dependent fluorescence signal increase due to their autofluorescent properties, which interfered with the ThO signal and produced distorted dose-response curves. One of the most potent DNA binders among the tested HTS hits was phthalocyanine tetrasulfonate hydrate (NCGC00165867), with a ThO dye displacement IC_50_ of 0.631 µM, which is a very similar potency to that seen in the primary screening assay (Figure 4B). We note that the ThO assay reports primarily on DNA binders acting through intercalation, while potentially missing other types of DNA binders, such as those acting through the minor groove. However, promiscuous DNA binders which may have been missed by the ThO assay are likely to be flagged during the next profiling step, *i.e.* the test for inhibition of *E. coli* EndoIV, described below.

To probe the selectivity of the candidate APE1 inhibitors, we tested them against *E. coli* EndoIV [36], employing the same assay as used in the HTS. *E. coli* EndoIV, while exhibiting similar biochemical activities to APE1, such as AP site incision, has no sequence or structural homology to human APE1 [37], and thus serves as a tool for identifying broad-acting endonuclease inhibitors. Twenty-one compounds yielded full or partial concentration response curves against EndoIV (Table S1). Of these 21 compounds, 15 were already revealed as DNA binders by the ThO assay, and thus, were anticipated to represent non-specific inhibitors. Of the EndoIV-positive hits, only six (compounds NCGC00024246, NCGC00094813, NCGC00159344, NCGC00161415, NCGC00165867, NCGC00166058, Table S1) possessed complete concentration response curves for EndoIV, and the IC_50_ values of these compounds were significantly higher than the corresponding APE1 potencies. These results indicate that the majority of qHTS-output compounds more selectively inhibit APE1 than EndoIV, and the six compounds above represent potential starting points for the design of EndoIV-specific inhibitors.

To gain insight into the mode of action of the hits, we employed a displacement assay combined with fluorescence polarization (FP) detection to test the small molecule’s effect on the binding of APE1 to a version of the fluorogenic AP site-containing oligonucleotide substrate that was devoid of the quencher functionality. FP is a convenient technique for providing a basic characterization of macromolecular associations, such as protein-DNA [38] and protein-protein interactions [39]. Following incubation of the compound, APE1, and DNA substrate together, inhibition of APE1 binding to AP-DNA would be revealed as a decrease in FP of the fluorophore. The assay, which is performed in the absence of magnesium to prevent enzymatic turnover, is target DNA-specific, as APE1 binds with higher affinity to duplex DNA containing an AP site than to an undamaged counterpart [36]. Of the 223 hits validated in the electrophoretic separation assays, 70 compounds showed a concentration-dependent decrease in FP in the displacement assay, indicating an inhibition of APE1 AP-DNA binding. These results provide an early indication with respect to the mechanism of action of each compound, although the sensitivity of the displacement assay is lower than that of the enzymatic assay, making it difficult to reveal relevant, but weak, protein binders.

### Potentiation of the Genotoxic Effect of MMS in HeLa Cells

To examine the biological prospects of the top compounds, we tested their ability to inhibit APE1 DNA repair activity by assessing enhancement of MMS toxicity in mammalian cells. MMS creates methylated-base damage to genomic DNA, which, when excised by an alkylpurine DNA glycosylase, results in a high number of cytotoxic AP sites [40]. We compared the viability of HeLa cells exposed to a dilution series of inhibitor alone to a combination of a fixed MMS concentration with the same dilution series of inhibitor by using a standard CellTiter Glo luminescence assay. This technique measures the number of viable cells in culture based on quantitation of the ATP present, which signals the presence of metabolically active cells. The assay was further optimized for 384-well plates, allowing a high-throughput testing of multiple hits at multiple concentrations, a scope of investigation previously impossible due to the very low throughput of the traditionally applied colony formation assay.

From the 223 compounds found positive in the gel-based APE1 assays, exclusion of the most promiscuous hits, that is, those that were both DNA binders and EndoIV-inhibitory, yielded a set of 170 compounds (Figure 3). This shortened group of hits was tested in the cell-based potentiation assay for their ability to dose-dependently enhance sensitivity of HeLa cells to the alkylating agent MMS. Of the 170 compounds tested, 12 enhanced the cytotoxicity of MMS significantly (the difference between the compound-plus-MMS and compound-alone responses was at least 30% and was maintained within at least a two-fold compound concentration range, Figure 5), while 16 compounds produced a modest response (the separation between the curves was greater than the errors of measurement but was less than 30% viability at any given compound concentration, Figure 6). The structures and assay data associated with the 12 compounds that exhibited significant effect in the MMS potentiation assay are presented in Figure 7.

### AP Site Accumulation

As stated above, the application of the genotoxic agent MMS results in an increase in the level of AP sites within the genome. An effective APE1-targeted inhibitor applied under the conditions of increased genotoxic stress is expected to interfere with the repair of AP sites, while ideally having no effect on the AP site levels when applied as a single agent. Thus, the net effect of treating cells with a combination of MMS and inhibitor would be an increase in the number of AP site lesions. To determine the effect on AP site accumulation caused by treatment with MMS in combination with an APE1 inhibitor, as compared to treatment with MMS or inhibitor alone, we measured AP site levels in the chromosomal DNA from HeLa cells using an aldehyde-reactive probe-based colorimetric assay [40]. The concentrations of MMS and inhibitor (275 µM for MMS and between 5 and 30 µM for the inhibitor, respectively) were selected to fall below the onset of cytotoxicity for each agent based on the analysis of the MMS potentiation data shown in Figure 5.

Of the 12 compounds that significantly enhanced the cytotoxicity of MMS (Figure 3), 11 were tested in the AP site accumulation experiments (MLS001217741 was not tested due to resupply shortage). These experiments revealed that in general the number of AP sites was only slightly increased in the cells exposed to the compounds alone relative to the vehicle control, but was significantly increased when cells were exposed to both MMS and most of the tested compounds (Figure 8). The exceptions to this trend were inhibitors where a noticeable increase in AP sites was observed upon treatment with the inhibitor alone (MLS001306480 (P = 0.0198), MLS001196838 (P = 0.0238), MLS001076862 (P = 0.0234)). Thus, compounds whose application as single agents resulted in an increase inAP sites may be of lesser utility as drug candidates, due to their potential to induce genotoxic damage by themselves. Also, for several hits, the combination treatment raised the level of AP sites very slightly (relatively small standard deviations and no statistical significance between AP site levels detected in combination treatments versus MMS-alone: MLS000737267, MLS000863573, MLS000594187, MLS001076862) or the increase in AP site levels with the combined treatment was obscured by large standard deviations observed in the combination experimental group (MLS000587064, MLS000090966, and MLS000115025). In an attempt to provide further support for the preliminary trends, we ran additional AP-site measurement experiments on the three hits (MLS000587064, MLS000090966, and MLS000115025) which produced the large error bars seen in the “Combined” treatment category (Figure 8): the repeat tests produced smaller standard deviations and appear to support the initially-observed enhancements for MLS000587064 and MLS000115025 (Figure S1). We note that because the AP site experiments are laborious in nature, and consistent with the need to provide initial profiling on a relatively large number of screening hits, the number of replicates incorporated in this experiment (two), as well as the combinations of inhibitor and MMS concentrations, were insufficient for a complete statistical analysis of the trends. In order for a true Combination Index to be derived [41], which in turn would allow for a definitive conclusion to be draw regarding the potential synergistic nature of the inhibitor effect on the MMS genotoxicity, a large matrix of inhibitor and MMS concentrations needs to be tested, making it impossible to provide such an exhaustive characterization for all top hits reported here. We further note that given the laborious nature of the AP site experiments, we have limited this initial study to only the 24-hour treatment condition: the longer-term effect (>3 days of treatment) of APE1 inhibitors on AP site accumulation is an important factor to consider during the development of one or more of the hits, particularly given the previously-highlighted vital role of APE1 as a resolver of abasic DNA damage accrued through natural causes [12].

With the exception of MLS001306480, an antimalarial pyronaridine used primarily in China [42], which is a quinacrine-like molecule that bears distant resemblance to the previously published APE1 inhibitors lucanthone [43], mitoxanthrone [21], and Reactive Blue 2 [21], none of the hit compounds that caused an increase in AP sites in cells under genotoxic stress were similar to previously reported APE1 inhibitors, including AR03 [23] or the pharmacophore model advanced by Zawahir and colleagues [22]. This overall result provides a support to the notion that screening of additional libraries to find novel APE1 inhibitory scaffolds is indeed a viable approach.

Of the top hits, substituted isoxazole alkylamines like MLS001105846 have been patented as agri-horticultural fungicides [44]. Although outside this current profiling work, the MLS001105846 compound does not appear to have been tested in conjunction with human disease applications. MLS000419194 possesses no obvious liabilities and was recently taken through a medicinal chemistry optimization campaign by us [45].

The remaining hits that potentiate the MMS response and cause AP site accumulation belong to structural classes typically associated with promiscuity (that is, prone to bind to multiple protein targets in the cell) or carry potentially reactive or labile functional groups. The flavonoid derivative MLS000737267, also known as galloflavin (a product derived from gallic acid oxidation), has been shown to inhibit human immunodeficiency virus integrase at a low-micromolar IC_50_ [46]. Phenanthrenes derived from orchid plant species, bearing similar chemical features to the trimethoxyphenanthrene-diol hit MLS000863573, have recently been highlighted for their anti-inflammatory activity, presumably by inhibiting the lipopolysacharide-induced nitric oxide production in murine macrophages [47]. Both MLS000737267 and MLS000863573 are undesirable from the standpoint of further medicinal chemistry optimization because of the polyphenolic nature. MLS000594187, which bears a resemblance to the above two hits, in that it has a core consisting of three fused 6-membered rings in a phenanthrene-like configuration, belongs to a different chemical class, benzo(c)chromen-6-ones, which has been advanced as selective estrogen receptor β modulators [48]. MLS001196838 was recently characterized as a potent and selective inhibitor of the histone acetyltransferase (HAT) p300, and has been employed to probe HAT’s role in acute myelogenous leukemia [49], [50]. A derivative of MLS001076862 has been reported as a modulator of the survival protein MCL-1, and displays cellular activity and thus potential utility for treating hyperproliferative, inflammatory, and other disorders [51]. Of note, MLS001196838 and MLS001076862 contain an exocyclic enone functionality, which may be reactive and would need to be modified prior to an extended optimization of these scaffolds. The pyrimidine-2,4,6-trione central core unit within MLS000587064 derives from barbituric acid, although this compound itself does not belong to the group of typical barbiturates. Most recently, pyrimidine-2,4,6-trione derivatives were shown to be highly effective in a protein aggregation protection assay model of amyotrophic lateral sclerosis and to possess good bioavailability [52]. Lastly, the furanyl-tetrahydromethyl-indole MLS000090966 was recently reported to be an effective inhibitor of tubulin assembly [53]. MLS000587064 and MLS000090966 contain a catechol diether moiety, a recognized chemical liability [54].

Taken together, our analyses have revealed a promising set of structurally diverse heretofore unreported APE1 inhibitors. As with all typical high-throughput screening and follow-up campaigns, it is plausible that additional APE1-inhibitory chemotypes of drug-like characteristics, which reside in our collection, may have been missed due to the application of the series of stringent selection criteria in order to narrow down the list of hits being followed up. At present, it is hoped that the public availability of all screening and secondary assay data will lead to further exploration of this information-rich resource.

## Supporting Information

Figure S1 Repeat AP site testing of the three hits whose combination treatments produced large standard deviations (MLS000587064, MLS000090966, and MLS000115025 in Figure 8) upon initial testing. A decrease in the standard deviation was observed for these compounds upon repeat testing, with significant difference (*P<0.05, **P<0.01) between the combination and MMS-alone treatments obtained for MLS000587064 and MLS000115025, confirming the initially-observed enhancement trends.
(EPS)

Table S1 HTS hits which displayed inhibition in the electrophoretic separation based assays.
(DOCX)
