# Supplementary material for: Diverse Small Molecule Inhibitors of Human Apurinic/Apyrimidinic Endonuclease APE1 Identified from a Screen of a Large Public Collection
Source: PLoS One. 2012 Oct 23;7(10):e47974. doi: 10.1371/journal.pone.0047974 (PMC3479139; doi:10.1371/journal.pone.0047974)
Supplement: Table S1 — HTS hits which displayed inhibition in the electrophoretic separation based assays. (DOCX) [file pone.0047974.s002.docx]

**Dorjsuren, et al., Supplemental Table S1.**

HTS hits which displayed inhibition in the electrophoretic separation based assays with detection of radiotracer (first 112 entries, percent incision shown) or fluorophore (entries 113-223, estimated IC_50_ in μM shown). Listed are: IC_50_ (μM) obtained in the initial quantitative high-throughput screen and upon a retest of cherrypicked sample; ThO-DNA binding, Endo IV, FP: IC_50_ (μM) or annotation of response (N.A., no activity observed; I, partial/incomplete concentration response curve); MMS: potentiation of the genotoxic effect of methylmethane sulfonate (N.A., no activity observed; P, positive; I, inconclusive; N.T., not tested).

| **No.** | **Substance ID** | **Radiotracer Assay (%Activity)** | **APE1 qHTS IC_50_ (μM)** | **APE1 Retest IC_50_ (μM)** | **ThO-DNA binding IC_50_ (μM)** | **EndoIV IC_50_ (μM)** | **FP IC_50_ (μM)** | **MMS**  **Assay** |
| --- | --- | --- | --- | --- | --- | --- | --- | --- |
| **1** | MLS000540800 | 0 | 8.9 | 12.6 | N.A. | N.A. | 15.8 | N.A. |
| **2** | MLS000530638 | 1 | 7.0 | 1.6 | 10 | N.A. | 8.9 | N.T. |
| **3** | MLS000627094 | 2 | 11.2 | 12.6 | N.A. | N.A. | 15.8 | N.A. |
| **4** | MLS000419194 | 2 | 14.1 | 3.9 | N.A. | 12.6 | 2.5 | P |
| **5** | NCGC00024246 | 2.3 | 14.1 | 12.6 | N.A. | 31.6 | 2.0 | N.T. |
| **6** | NCGC00094975 | 2.4 | 0.01 | 0.01 | 0.1 | N.A. | 31.6 | N.T. |
| **7** | NCGC00163605 | 2.7 | 2.0 | 1.6 | 12.6 | N.A. | 2.2 | N.T. |
| **8** | NCGC00094813 | 2.8 | 25.1 | 6.4 | N.A. | 10 | 15.8 | N.T. |
| **9** | MLS000778775 | 3 | 19.9 | 12.6 | N.A. | N.A. | 5.0 | N.A. |
| **10** | MLS000113273 | 3 | 14.1 | 3.2 | N.A. | N.A. | N.A. | N.A. |
| **11** | MLS000863573 | 4 | 0.7 | 0.6 | 11.2 | 8.9 | 12.6 | P |
| **12** | NCGC00095664 | 4.1 | 0.1 | 0.1 | 11.2 | N.A. | 15.8 | N.T. |
| **13** | NCGC00159344 | 4.4 | 25.1 | 8.9 | N.A. | 12.6 | 1.6 | N.T. |
| **14** | NCGC00093976 | 4.6 | 19.9 | 14.1 | N.A. | 25.1 | 3.2 | N.T. |
| **15** | NCGC00161415 | 4.6 | 4.5 | 10.0 | 14.1 | 12.6 | 1.3 | N.T. |
| **16** | MLS000677191 | 5 | 14.1 | 4.5 | N.A. | N.A. | N.A. | I |
| **17** | MLS000778776 | 5 | 25.1 | 12.39 | N.A. | 4.5 | 0.02 | N.T. |
| **18** | MLS000778639 | 6 | 2.5 | 3.5 | N.A. | N.A. | N.A. | I |
| **19** | MLS000621695 | 6 | 2.8 | 1.6 | 15.8 | N.A. | 11.2 | N.T. |
| **20** | MLS000699310 | 6 | 31.6 | 5.0 | N.A. | N.A. | N.A. | N.A. |
| **21** | NCGC00165867 | 6.2 | 0.3 | 0.06 | 0.6 | 1.6 | 0.2 | N.T. |
| **22** | MLS000704580 | 7 | 17.8 | 11.2 | N.A. | N.A. | N.A. | N.A. |
| **23** | MLS000689657 | 7 | 25.1 | 12.6 | 14.1 | N.A. | N.A. | N.T. |
| **24** | MLS000574902 | 8 | 0.01 | 0.006 | 4.0 | N.A. | N.A. | N.T. |
| **25** | NCGC00163667 | 8.4 | 10.0 | 4.4 | 10 | N.A. | N.A. | N.T. |
| **26** | MLS000551724 | 9 | 2.8 | 0.9 | N.A. | N.A. | 12.6 | I |
| **27** | MLS000039532 | 9 | 22.4 | 7.9 | N.A. | N.A. | N.A. | N.A. |
| **28** | MLS000737782 | 9 | 14.1 | 15.8 | N.A. | N.A. | N.A. | N.A. |
| **29** | MLS000759952 | 10 | 10 | 12.6 | N.A. | N.A. | N.A. | N.A. |
| **30** | MLS000697684 | 10 | 25.1 | 12.6 | N.A. | N.A. | N.A. | N.A. |
| **31** | NCGC00165741 | 10.6 | 3.2 | 0.3 | N.A. | N.A. | N.A. | N.T. |
| **32** | MLS000107874 | 11 | 22.4 | 8.9 | N.A. | N.A. | 17.8 | I |
| **33** | MLS000534769 | 11 | 19.9 | 4.0 | 8.9 | 10 | N.A. | N.T. |
| **34** | NCGC00093869 | 11.3 | 1.00 | 3.5 | N.A. | N.A. | 31.6 | N.T. |
| **35** | MLS000548032 | 13 | 14.1 | 12.6 | N.A. | N.A. | N.A. | N.T. |
| **36** | NCGC00166058 | 13.4 | 31.6 | 25.1 | 35.5 | 28.2 | 31.6 | N.T. |
| **37** | MLS000587064 | 14 | 2.8 | 5.0 | N.A. | N.A. | N.A. | P |
| **38** | MLS000062734 | 14 | 10 | 5.6 | N.A. | N.A. | N.A. | N.A. |
| **39** | MLS000092582 | 14 | 31.6 | 10 | N.A. | N.A. | N.A. | I |
| **40** | MLS000686355 | 15 | 3.5 | 1.4 | N.A. | N.A. | 14.1 | N.A. |
| **41** | MLS000563483 | 16 | 0.07 | 0.05 | N.A. | N.A. | N.A. | I |
| **42** | MLS001005620 | 17 | 11.2 | 4.4 | N.A. | N.A. | N.A. | N.T. |
| **43** | MLS000737360 | 17 | 8.9 | 6.3 | 6.3 | N.A. | 7.9 | N.T. |
| **44** | NCGC00024700 | 17.1 | 1.8 | N.A. | N.A. | N.A. | N.A. | N.T. |
| **45** | MLS000723827 | 18 | 22.4 | 14.1 | N.A. | N.A. | N.A. | I |
| **46** | MLS000551672 | 19 | 1.6 | 2.5 | N.A. | N.A. | 11.2 | N.T. |
| **47** | MLS000763226 | 20 | 31.6 | 15.8 | 10 | N.A. | N.A. | I |
| **48** | MLS000768482 | 21 | 22.4 | 4.5 | N.A. | N.A. | N.A. | N.A. |
| **49** | MLS000559128 | 21 | 31.6 | 10 | N.A. | N.A. | 7.1 | N.T. |
| **50** | MLS000530771 | 21 | 19.9 | 7.1 | N.A. | N.A. | 10 | N.A. |
| **51** | MLS000069140 | 22.3 | 22.4 | 12.6 | N.A. | N.A. | N.A. | N.A. |
| **52** | MLS000777053 | 23 | 10 | 5.0 | 6.3 | N.A. | 7.9 | N.T. |
| **53** | MLS000703171 | 23 | 7.1 | 12.6 | N.A. | N.A. | N.A. | N.A. |
| **54** | MLS000879461 | 24 | 25.1 | 7.9 | N.A. | N.A. | 14.1 | N.A. |
| **55** | MLS000539676 | 25 | 19.9 | 14.1 | N.A. | N.A. | N.A. | N.T. |
| **56** | NCGC00161652 | 26.5 | 7.9 | 2.5 | N.A. | N.A. | 31.6 | N.T. |
| **57** | MLS000759946 | 27 | 2.5 | 14.1 | N.A. | N.A. | 15.8 | N.T. |
| **58** | MLS000685729 | 28 | 31.6 | 5.0 | N.A. | N.A. | N.A. | N.T. |
| **59** | MLS000535286 | 28 | 11.2 | 12.6 | N.A. | N.A. | N.A. | I |
| **60** | MLS000713642 | 28 | 12.6 | 7.9 | N.A. | N.A. | 15.8 | N.T. |
| **61** | NCGC00095195 | 28.3 | 0.3 | 0.1 | N.A. | N.A. | N.A. | N.T. |
| **62** | MLS000697665 | 29 | 0.01 | 0.01 | 0.4 | N.A. | 14.1 | N.T. |
| **63** | MLS000088142 | 29 | 15.6 | 5.0 | N.A. | N.A. | 14.1 | I |
| **64** | MLS000876823 | 30 | 3.5 | 3.1 | N.A. | N.A. | N.A. | N.T. |
| **65** | MLS000673948 | 30 | 3.5 | 4.4 | N.A. | N.A. | N.A. | N.T. |
| **66** | MLS000567201 | 31 | 14.1 | 3.2 | 15.8 | N.A. | N.A. | N.T. |
| **67** | MLS000876755 | 31 | 19.9 | 4.5 | N.A. | N.A. | N.A. | N.T. |
| **68** | MLS000676588 | 31 | 5.6 | 8.9 | N.A. | N.A. | N.A. | N.A. |
| **69** | MLS000711532 | 32 | 28.2 | 10 | N.A. | N.A. | N.A. | N.A. |
| **70** | MLS000588622 | 32 | 5.0 | 3.5 | N.A. | N.A. | N.A. | N.A. |
| **71** | MLS000621664 | 32 | 17.8 | 12.6 | N.A. | N.A. | N.A. | N.A. |
| **72** | NCGC00161639 | 32.8 | 10.0 | 7.9 | N.A. | N.A. | N.A. | N.T. |
| **73** | MLS000685551 | 33 | 19.9 | 12.6 | N.A. | N.A. | N.A. | N.A. |
| **74** | MLS000807042 | 33 | 1.1 | 14.1 | N.A. | N.A. | N.A. | N.A. |
| **75** | MLS000548444 | 35 | 12.6 | 12.6 | N.A. | 10 | 15.8 | N.A. |
| **76** | NCGC00095270 | 35.9 | 3.2 | 2.8 | N.A. | N.A. | N.A. | N.T. |
| **77** | MLS000673045 | 36 | 12.6 | 4.5 | 3.2 | 14.1 | N.A. | N.A. |
| **78** | MLS001004396 | 36 | 8.9 | 8.9 | N.A. | N.A. | N.A. | N.A. |
| **79** | MLS000762704 | 36 | 4.5 | 4.5 | N.A. | N.A. | N.A. | N.A. |
| **80** | MLS000534059 | 36 | 14.1 | 8.9 | N.A. | N.A. | N.A. | N.T. |
| **81** | MLS000876981 | 36 | 8.9 | 11.2 | N.A. | N.A. | N.A. | N.T. |
| **82** | NCGC00094525 | 36.6 | 20.0 | 44.7 | N.A. | N.A. | N.A. | N.T. |
| **83** | MLS000090966 | 37 | 7.1 | 4.5 | N.A. | N.A. | 15.8 | P |
| **84** | MLS000120697 | 37 | 12.6 | 3.9 | N.A. | N.A. | 7.9 | N.A. |
| **85** | MLS000778414 | 37 | 2.5 | 4.0 | N.A. | N.A. | 12.6 | N.A. |
| **86** | MLS000879482 | 37 | 22.4 | 5.0 | N.A. | N.A. | 12.6 | N.T. |
| **87** | MLS000682903 | 37 | 12.6 | 15.6 | N.A. | N.A. | 15.8 | N.T. |
| **88** | MLS001049122 | 37 | 0.4 | 6.3 | N.A. | N.A. | N.A. | N.T. |
| **89** | NCGC00160279 | 37.9 | 1.1 | 0.4 | 1.26 | N.A. | N.A. | N.T. |
| **90** | MLS000594161 | 38 | 11.2 | 5.6 | N.A. | N.A. | N.A. | N.A. |
| **91** | MLS000697677 | 38 | 0.1 | 0.6 | N.A. | N.A. | N.A. | N.T. |
| **92** | MLS000334872 | 39 | 156 | 10 | N.A. | N.A. | 12.6 | N.A. |
| **93** | MLS000114057 | 40 | 4.0 | 17.8 | N.A. | N.A. | N.A. | N.A. |
| **94** | MLS000778631 | 41 | 35.5 | 5.0 | N.A. | N.A. | N.A. | N.A. |
| **95** | MLS000050353 | 41 | 3.5 | 4.0 | N.A. | 15.8 | 12.6 | N.T. |
| **96** | NCGC00094863 | 41 | 22.4 | N.A. | N.A. | N.A. | N.A. | N.T. |
| **97** | MLS000556463 | 43 | 15.6 | 3.6 | N.A. | N.A. | N.A. | N.A. |
| **98** | MLS000877008 | 43 | 1.1 | 2.8 | N.A. | N.A. | N.A. | I |
| **99** | MLS000120556 | 43 | 14.1 | 5.0 | N.A. | N.A. | N.A. | N.T. |
| **100** | MLS000391557 | 44 | 17.8 | 6.3 | N.A. | N.A. | N.A. | I |
| **101** | MLS000876957 | 44 | 1.6 | 4.5 | N.A. | N.A. | N.A. | N.A. |
| **102** | MLS000621431 | 44 | 11.2 | 5.0 | N.A. | N.A. | N.A. | N.T. |
| **103** | MLS000715767 | 44 | 25.1 | 14.1 | 11.2 | N.A. | 12.6 | N.T. |
| **104** | MLS000760918 | 45 | 14.1 | 15.8 | N.A. | N.A. | N.A. | N.A. |
| **105** | MLS000778420 | 46 | 3.9 | 4.5 | N.A. | N.A. | N.A. | P |
| **106** | MLS000737267 | 46 | 0.4 | 0.13 | 6.3 | N.A. | N.A. | P |
| **107** | MLS001006993 | 47 | 22.4 | 14.1 | N.A. | N.A. | N.A. | N.A. |
| **108** | MLS000759943 | 47 | 8.9 | 10 | N.A. | N.A. | N.A. | N.T. |
| **109** | MLS000115025 | 48 | 28.2 | 2.0 | N.A. | N.A. | N.A. | P |
| **110** | MLS000850015 | 49 | 11.2 | 15.8 | N.A. | N.A. | N.A. | N.A. |
| **111** | MLS000685676 | 50 | 14.1 | 14.1 | N.A. | N.A. | N.A. | N.A. |
| **112** | MLS000594187 | 51 | 10 | 3.91 | N.A. | N.A. | N.A. | P |
| **No.** | **Substance ID** | **F-PAGE Assay IC_50_ (μM)** | **APE1 qHTS IC_50_ (μM)** | **APE1 Retest IC_50_ (μM)** | **ThO-DNA binding IC_50_ (μM)** | **EndoIV IC_50_ (μM)** | **FP IC_50_ (μM)** | **MMS**  **Assay** |
| **113** | MLS001081306 | 0.1 | 1.9 | 7.3 | 40.8 | N.A. | N.A. | N.A. |
| **114** | MLS001233727 | 0.1 | 0.1 | 0.3 | N.A. | N.A. | N.A. | N.A. |
| **115** | MLS001080179 | 0.3 | 0.8 | 1.1 | N.A. | N.A. | N.A. | N.A. |
| **116** | MLS001173932 | 0.6 | 3.0 | 5.1 | 2.0 | N.A. | N.A. | I |
| **117** | MLS002159357 | 0.6 | 2.1 | 10.2 | N.A. | N.A. | N.A. | N.A. |
| **118** | MLS000974607 | 0.7 | 1.9 | 5.1 | N.A. | N.A. | N.A. | N.A. |
| **119** | MLS002152987 | 0.9 | 0.5 | 5.1 | N.A. | N.A. | N.A. | N.A. |
| **120** | MLS001139292 | 1.0 | 10.6 | 6.5 | N.A. | N.A. | N.A. | N.A. |
| **121** | MLS001141795 | 1.1 | 1.9 | 6.5 | N.A. | N.A. | N.A. | N.A. |
| **122** | MLS001178474 | 1.4 | 6.7 | 5.1 | 10.2 | N.A. | N.A. | N.A. |
| **123** | MLS002171184 | 1.6 | 3.4 | 11.5 | 32.4 | N.A. | N.A. | N.A. |
| **124** | MLS002248682 | 2.0 | 2.7 | 8.1 | N.A. | N.A. | N.A. | N.A. |
| **125** | MLS001142042 | 2.2 | 4.7 | 14.5 | N.A. | N.A. | N.A. | I |
| **126** | MLS001076862 | 4.0 | 10.6 | N.A. | N.A. | N.A. | N.T. | P |
| **127** | MLS000738229 | 2.5 | 13.4 | 3.6 | 28.9 | N.A. | N.A. | N.A. |
| **128** | MLS001079471 | 2.5 | 4.2 | 11.5 | N.A. | N.A. | 10.2 | N.A. |
| **129** | MLS001139223 | 2.5 | 1.9 | 2.0 | N.A. | N.A. | N.A. | N.A. |
| **130** | MLS001139594 | 2.5 | 1.5 | 11.5 | N.A. | N.A. | N.A. | N.A. |
| **131** | MLS001194759 | 2.5 | 2.7 | 3.2 | 10.2 | N.A. | N.A. | N.A. |
| **132** | MLS000948181 | 2.8 | 13.4 | 20.4 | N.A. | N.A. | N.A. | N.A. |
| **133** | MLS001098232 | 2.8 | 6.7 | 6.5 | N.A. | N.A. | 20.4 | N.A. |
| **134** | MLS001159279 | 2.8 | 2.7 | 12.9 | N.A. | N.A. | N.A. | N.A. |
| **135** | MLS001161412 | 2.8 | 3.0 | 5.8 | N.A. | 16.2 | 12.9 | N.A. |
| **136** | MLS002164959 | 2.8 | 1.3 | 11.5 | N.A. | N.A. | N.A. | N.A. |
| **137** | MLS002222332 | 2.8 | 11.9 | 0.0 | N.A. | N.A. | N.A. | N.A. |
| **138** | MLS001060910 | 3.2 | 11.9 | 18.2 | N.A. | N.A. | N.A. | N.A. |
| **139** | MLS000738241 | 3.5 | 7.5 | 12.9 | N.A. | N.A. | N.A. | N.A. |
| **140** | MLS001060429 | 3.5 | 10.6 | 9.1 | N.A. | N.A. | N.A. | N.A. |
| **141** | MLS001097639 | 3.5 | 2.4 | 18.2 | N.A. | N.A. | N.A. | N.A. |
| **142** | MLS002162303 | 3.5 | 10.6 | 9.1 | N.A. | N.A. | N.A. | N.A. |
| **143** | MLS002251079 | 3.5 | 3.0 | 12.9 | N.A. | N.A. | 18.2 | N.A. |
| **144** | MLS001099510 | 3.5 | 5.3 | 22.9 | N.A. | N.A. | N.A. | N.A. |
| **145** | MLS001128172 | 4.0 | 1.7 | 20.4 | N.A. | N.A. | N.A. | N.A. |
| **146** | MLS001156641 | 4.0 | 10.6 | 36.3 | 45.7 | N.A. | N.A. | N.A. |
| **147** | MLS001175210 | 4.0 | 7.5 | 2.9 | N.A. | N.A. | 20.4 | N.A. |
| **148** | MLS002161511 | 4.0 | 6.0 | 6.5 | N.A. | 18.2 | 8.1 | N.A. |
| **149** | MLS002245402 | 4.0 | 8.4 | 18.2 | 32.4 | N.A. | N.A. | N.A. |
| **150** | MLS002251323 | 4.0 | 9.5 | 9.1 | N.A. | N.A. | N.A. | N.A. |
| **151** | MLS002252586 | 4.0 | 7.5 | 14.5 | N.A. | N.A. | N.A. | N.A. |
| **152** | MLS001105846 | 4.5 | 11.9 | 6.5 | N.A. | N.A. | 32.4 | P |
| **153** | MLS002252117 | 4.5 | 5.3 | 11.5 | N.A. | N.A. | N.A. | I |
| **154** | MLS001161521 | 4.5 | 3.8 | 7.3 | N.A. | N.A. | N.A. | N.A. |
| **155** | MLS001071694 | 5.0 | 3.4 | 5.1 | N.A. | N.A. | 22.9 | N.A. |
| **156** | MLS002252055 | 5.0 | 8.4 | 6.5 | 40.8 | N.A. | N.A. | N.A. |
| **157** | MLS001234358 | 5.0 | 11.9 | 11.5 | N.A. | 36.3 | 18.2 | N.A. |
| **158** | MLS001008046 | 5.6 | 5.3 | 8.1 | N.A. | N.A. | 28.9 | N.A. |
| **159** | MLS001030262 | 5.6 | 29.9 | 12.9 | N.A. | N.A. | N.A. | N.A. |
| **160** | MLS001175210 | 5.6 | 7.5 | 2.9 | N.A. | N.A. | 20.4 | N.A. |
| **161** | MLS002320105 | 5.6 | 11.9 | N.A. | N.A. | N.A. | N.A. | N.A. |
| **162** | MLS000974674 | 6.3 | 11.9 | 45.7 | 36.3 | N.A. | N.A. | P |
| **163** | MLS001156482 | 6.3 | 7.5 | 36.3 | 40.8 | N.A. | N.A. | N.A. |
| **164** | MLS002169032 | 6.3 | 10.6 | 10.2 | N.A. | N.A. | N.A. | N.A. |
| **165** | MLS001209038 | 6.3 | 13.4 | 18.2 | 25.7 | N.A. | N.A. | I |
| **166** | MLS001077576 | 7.1 | 8.4 | 14.5 | N.A. | N.A. | 32.4 | N.A. |
| **167** | MLS001196440 | 7.1 | 9.5 | 7.3 | 40.8 | N.A. | N.A. | N.A. |
| **168** | MLS001211293 | 7.1 | 13.4 | N.A. | N.A. | N.A. | N.A. | N.A. |
| **169** | MLS001234379 | 7.1 | 10.6 | 11.5 | N.A. | N.A. | N.A. | N.A. |
| **170** | MLS002159150 | 7.1 | 5.3 | 11.5 | N.A. | N.A. | 36.3 | N.A. |
| **171** | MLS002164354 | 7.1 | 11.9 | 14.5 | N.A. | 22.9 | 36.3 | N.A. |
| **172** | MLS002251505 | 7.1 | 6.7 | 9.1 | N.A. | N.A. | N.A. | N.A. |
| **173** | MLS002169571 | 7.1 | 10.6 | 45.7 | N.A. | N.A. | N.A. | N.A. |
| **174** | MLS001163009 | 7.9 | 11.9 | 12.9 | N.A. | N.A. | 25.7 | N.A. |
| **175** | MLS001194971 | 7.9 | 21.2 | 18.2 | N.A. | N.A. | N.A. | N.A. |
| **176** | MLS001196838 | 7.9 | 8.4 | 25.7 | 16.2 | N.A. | N.A. | P |
| **177** | MLS001216111 | 7.9 | 16.8 | 32.4 | 36.3 | N.A. | N.A. | N.A. |
| **178** | MLS002171489 | 7.9 | 8.4 | 16.2 | N.A. | N.A. | N.A. | N.A. |
| **179** | MLS002320191 | 7.9 | 6.7 | 20.4 | N.A. | N.A. | N.A. | N.A. |
| **180** | MLS001156783 | 7.9 | 10.6 | 10.2 | N.A. | N.A. | N.A. | N.A. |
| **181** | MLS001219746 | 8.9 | 21.2 | 16.2 | N.A. | N.A. | N.A. | N.A. |
| **182** | MLS002162311 | 8.9 | 10.6 | 32.4 | 36.3 | N.A. | N.A. | N.A. |
| **183** | MLS002246602 | 8.9 | 4.7 | 12.9 | N.A. | N.A. | 28.9 | N.A. |
| **184** | MLS002163634 | 8.9 | 10.6 | 10.2 | N.A. | N.A. | 18.2 | N.A. |
| **185** | MLS000999698 | 10.0 | 7.5 | 22.9 | N.A. | N.A. | 40.8 | N.A. |
| **186** | MLS001090278 | 10.0 | 5.3 | 10.2 | N.A. | N.A. | N.A. | I |
| **187** | MLS001359901 | 10.0 | 9.5 | 5.8 | N.A. | N.A. | N.A. | N.A. |
| **188** | MLS002156353 | 10.0 | 13.4 | 32.4 | 32.4 | N.A. | N.A. | N.A. |
| **189** | MLS001142077 | 10.0 | 10.6 | 32.4 | 20.4 | N.A. | N.A. | N.A. |
| **190** | MLS001001328 | 11.2 | 15.0 | 32.4 | N.A. | N.A. | N.A. | N.A. |
| **191** | MLS001178472 | 11.2 | 10.6 | 14.5 | N.A. | N.A. | N.A. | N.A. |
| **192** | MLS002163823 | 11.2 | 10.6 | 14.5 | N.A. | N.A. | 12.9 | N.A. |
| **193** | MLS002245326 | 11.2 | 10.6 | 4.6 | N.A. | N.A. | N.A. | N.A. |
| **194** | MLS002251710 | 11.2 | 26.7 | 16.2 | N.A. | N.A. | 20.4 | N.A. |
| **195** | MLS001159876 | 11.2 | 11.9 | 18.2 | 11.5 | N.A. | N.A. | N.A. |
| **196** | MLS001234358 | 11.2 | 11.9 | 11.5 | N.A. | 36.3 | 18.2 | N.A. |
| **197** | MLS001147809 | 12.6 | 10.6 | 14.5 | N.A. | N.A. | N.A. | N.A. |
| **198** | MLS001177378 | 12.6 | 26.7 | 45.7 | 32.4 | N.A. | N.A. | N.A. |
| **199** | MLS001235900 | 12.6 | 10.6 | 45.7 | N.A. | N.A. | 18.2 | N.A. |
| **200** | MLS001306480 | 12.6 | 15.0 | 6.5 | N.A. | 28.9 | 32.4 | P |
| **201** | MLS001202289 | 12.6 | 11.9 | 45.7 | 45.7 | N.A. | N.A. | N.A. |
| **202** | MLS001000225 | 14.1 | 13.4 | N.A. | N.A. | N.A. | 20.4 | I |
| **203** | MLS001139127 | 14.1 | 23.8 | 32.4 | N.A. | N.A. | N.A. | N.A. |
| **204** | MLS001234389 | 14.1 | 21.2 | 12.9 | N.A. | N.A. | N.A. | N.A. |
| **205** | MLS002181609 | 14.1 | 29.9 | 10.2 | N.A. | N.A. | 20.4 | N.A. |
| **206** | MLS001078387 | 14.1 | 13.4 | 32.4 | N.A. | N.A. | N.A. | N.A. |
| **207** | MLS002251953 | 15.8 | 10.6 | 14.5 | N.A. | N.A. | N.A. | N.A. |
| **208** | MLS001008562 | 17.8 | 13.4 | 45.7 | N.A. | N.A. | N.A. | N.A. |
| **209** | MLS002170765 | 17.8 | 15.0 | 18.2 | N.A. | N.A. | N.A. | N.A. |
| **210** | MLS001140219 | 20.0 | 13.4 | 18.2 | N.A. | N.A. | N.A. | N.A. |
| **211** | MLS001172702 | 20.0 | 11.9 | 14.5 | N.A. | N.A. | N.A. | N.A. |
| **212** | MLS002248300 | 20.0 | 21.2 | 32.4 | N.A. | N.A. | N.A. | N.A. |
| **213** | MLS002320206 | 20.0 | 15.0 | 16.2 | N.A. | N.A. | N.A. | N.A. |
| **214** | MLS002252767 | 20.0 | 5.3 | 14.5 | N.A. | N.A. | N.A. | N.A. |
| **215** | MLS001078987 | 22.4 | 16.8 | N.A. | N.A. | N.A. | 20.4 | N.A. |
| **216** | MLS001158971 | 22.4 | 21.2 | 9.1 | N.A. | N.A. | N.A. | N.A. |
| **217** | MLS001194618 | 22.4 | 29.9 | 14.5 | N.A. | N.A. | 36.3 | N.A. |
| **218** | MLS001217741 | 22.4 | 16.8 | 12.9 | N.A. | N.A. | N.A. | P |
| **219** | MLS002320455 | 22.4 | 9.5 | 10.2 | N.A. | N.A. | N.A. | N.A. |
| **220** | MLS001195935 | 25.1 | 18.9 | 18.2 | 32.4 | N.A. | N.A. | N.A. |
| **221** | MLS000737558 | 28.2 | 18.9 | 14.5 | N.A. | 36.3 | 20.4 | N.A. |
| **222** | MLS001081306 | 0.1 | 1.9 | 7.3 | 40.8 | N.A. | N.A. | N.A. |
| **223** | MLS001233727 | 0.1 | 0.1 | 0.3 | N.A. | N.A. | N.A. | N.A. |
